# Population Genetic Analysis of *Propionibacterium acnes* Identifies a Subpopulation and Epidemic Clones Associated with Acne

**DOI:** 10.1371/journal.pone.0012277

**Published:** 2010-08-19

**Authors:** Hans B. Lomholt, Mogens Kilian

**Affiliations:** Department of Medical Microbiology and Immunology, Aarhus University, Aarhus, Denmark; Charité-Universitätsmedizin Berlin, Germany

## Abstract

The involvement of *Propionibacterium* acnes in the pathogenesis of acne is controversial, mainly owing to its dominance as an inhabitant of healthy skin. This study tested the hypothesis that specific evolutionary lineages of the species are associated with acne while others are compatible with health. Phylogenetic reconstruction based on nine housekeeping genes was performed on 210 isolates of *P. acnes* from well-characterized patients with acne, various opportunistic infections, and from healthy carriers. Although evidence of recombination was observed, the results showed a basically clonal population structure correlated with allelic variation in the virulence genes *tly* and *camp5*, with pulsed field gel electrophoresis (PFGE)- and biotype, and with expressed putative virulence factors. An unexpected geographically and temporal widespread dissemination of some clones was demonstrated. The population comprised three major divisions, one of which, including an epidemic clone, was strongly associated with moderate to severe acne while others were associated with health and opportunistic infections. This dichotomy correlated with previously observed differences in *in vitro* inflammation-inducing properties. Comparison of five genomes representing acne- and health-associated clones revealed multiple both cluster- and strain-specific genes that suggest major differences in ecological preferences and redefines the spectrum of disease-associated virulence factors. The results of the study indicate that particular clones of *P. acnes* play an etiologic role in acne while others are associated with health.

## Introduction

The involvement of *Propionibacterium acnes* in the pathogenesis of acne has been controversial. It is debated whether *P. acnes* is the etiologic agent or if the observed therapeutic effect of certain antibiotics is due mainly to anti-inflammatory properties [1], [2]. Contributing to this uncertainty is that *P. acnes* is a dominant member of the resident microbiota of healthy human skin, and an exclusive bacterial inhabitant of normal human facial sebaceous follicles [3]. Nevertheless, several *in vitro* and *in vivo* studies show that *P. acnes* induces inflammatory responses in host keratinocytes, sebocytes, and monocytes and influences local cell growth and differentiation [4]–[14]. *P. acnes* is occasionally implicated in serious opportunistic infections such as endocarditis, osteomyelitis, and meningitis, and in severe, postsurgical infections after implantation of a foreign body, e.g., intraocular lenses, prosthetic heart valves, shunts, and posterior implants for scoliosis patients [15], [16].

An important recognition, resulting from advances in genetic analysis, is the considerable diversity that exists within bacterial species, and that particular subpopulations may be responsible for infections while others are non-pathogenic [17]–[19]. Like other bacterial species, *P. acnes* shows phenotypic and genotypic diversity. The species has been subdivided into three types, I–III and two subtypes IA and IB, initially based on serologic differentiation of cell wall carbohydrates and phage typing and later confirmed by analysis of *recA*, *tly*, and CAMP gene sequences [20]–[22]. In addition, biotyping based on fermentation reactions and typing by pulsed field gel electrophoresis (PFGE) suggests heterogeneity [23], [24]. Suggestive of differences in pathogenic potential, strains of *P. acnes* differ in their ability to induce human β-defensin 2 and to influence cell growth, differentiation and viability of keratinocytes and sebocytes [4]–[6].

This study was undertaken to determine the population structure of *P. acnes* with emphasis on isolates from skin, and to test the hypothesis that the species includes evolutionary lineages that live in harmony with their host and others that have the capacity to induce acne. Phylogenetic reconstruction and genetic analysis were performed on a collection of isolates from patients with defined grades of acne, from opportunistic infections, and from healthy carriers by multilocus sequence analysis (MLSA) based on nine housekeeping genes. The results were correlated with detailed clinical information, with allelic variation in selected virulence genes, phenotypic characteristics, and with results of PFGE typing. Gene contents of five representative complete and non-closed *P. acnes* genomes were compared to determine the contribution of variable genes to the genetic diversity within the species and to identify disease-associated properties.

## Results

### Sequence Polymorphism of Housekeeping and Virulence Genes

For each of 210 isolates identified as *P. acnes* according to appearance on agar plates, biochemical and physiological characteristics, and 16S rRNA gene sequence, nucleotide sequences were determined for internal fragments of each of nine housekeeping genes and two putative virulence factor genes *tly* and *camp5*. The size of the trimmed gene fragments varied from 363 bp (*cel*) to 807 bp (*camp5*). Comparison of aligned sequences revealed between six (*oxc*) and 15 (*coa*) alleles at the nine housekeeping gene loci and 12 and 11 in the two putative virulence genes *camp5* and *tly*, respectively. The genetic characteristics of the 11 gene loci are summarized in Table S1. The sequences are deposited at GenBank under accession numbers HQ003300–HQ003402.

### Population Structure Inferred from Concatenated Housekeeping Gene Sequences

**Figure 1:**
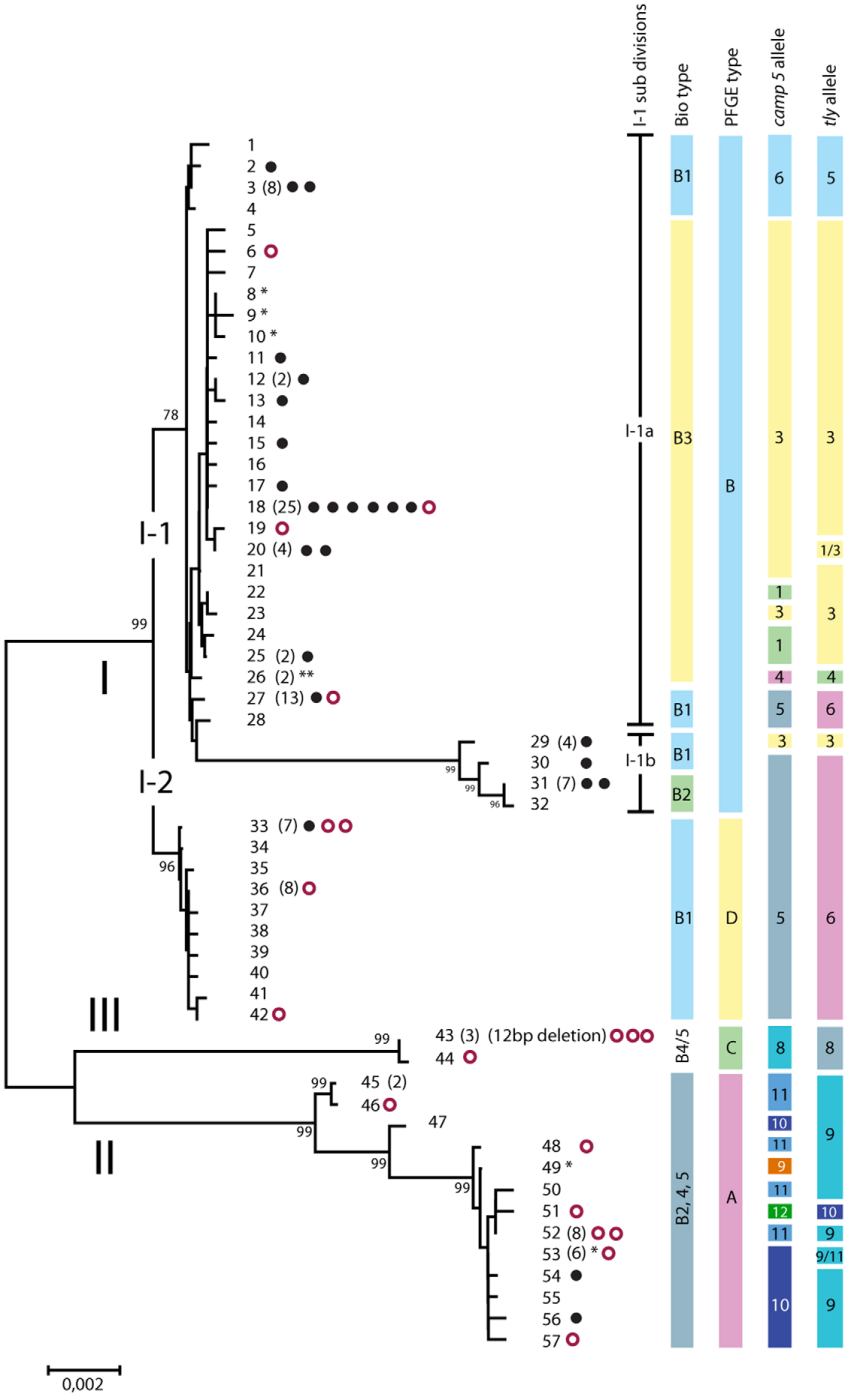
Population structure of *P. acnes*. Minimum evolution tree of 57 sequence types (ST) detected among 210 isolates of *P. acnes* from skin of healthy individuals, from patients with varying degrees of acne, and from other infectious diseases. The majority of isolates were from Caucasians. Isolates from healthy Chinese individuals are indicated by an asterix. The number of independent isolates assigned to each ST containing more than one isolate is shown in brackets. Each isolate from cases of severe acne is indicated by a filled black circle and isolates from infections in normally sterile tissues and blood are indicated by an open red circle. The correlation of clusters with biotype, PFGE type, and *tly* and *camp5* alleles is shown to the right. The bar represents the genetic distance.

Multilocus sequence analysis (MLSA) based on the concatenated sequences of the nine housekeeping genes (4287 bp in total) identified 57 sequence types (STs) among the 210 isolates with an overall mean genetic distance of 0.01±0.001. For the final construction of a phylogenetic tree and calculations of frequencies only one representative of isolates with identical ST from the same person was included, leaving 143 isolates. Individual STs were represented by from 25 (ST18) to single isolates (Figure 1). Twenty isolates from Chinese individuals represented 7 STs (indicated by an asterix in Figure 1). One was part of ST53 together with 5 isolates from Denmark and Sweden, and the remaining were distinct but deviated from neighbouring Danish isolates only by 1 to 4 substitutions in the 4287-bp concatenated sequence.

Three major clusters termed divisions I, II, and III, each supported by a bootstrap value of 99 encompassed a total of 113, 26, and 4 independent isolates. Division I was further divided into two subdivisions, I-1 and I-2, supported by bootstrap values of 78 and 96 (75 corresponds to statistical significance at the 0.05 level), and including 90 and 23 isolates, respectively. Among the 90 subdivision I-1 isolates, 13 segregated from the remaining 77 into a distinct cluster, termed I-1b. The 13 isolates of cluster I-1b shared nine substitutions in the *cel* sequence that were unique to that cluster and accounted for its segregation from I-1a. The allele profiles of the 57 STs in the nine housekeeping and two virulence genes are summarized in Table S2

### Virulence Gene Sequences

The degree of genetic diversity among *camp5* and *tly* genes did not deviate from that of housekeeping genes assumed to be selectively neutral (Table S1). A generally strong association of the *camp5* and *tly* alleles with the clustering based on concatenated housekeeping gene sequences was observed (Figure 1), the most striking exceptions being STs 27 and 28 of cluster I-1a and STs 30, 31, and 32 of cluster I-1b, which had *camp5* and *tly* alleles identical to strains of subdivision I-2. More pronounced *camp5* and *tly* gene polymorphism was found among division II strains than among strains of other divisions.

### Evidence of Limited Recombination

Several analyses were performed to evaluate the impact of recombination on the shaping of genomes of *P. acnes*. Theoretically, phylogenetic reconstructions based on different housekeeping genes must result in concordant trees in a clonal population in which accumulation of mutations is solely responsible for genetic diversification. Therefore, our first approach was to construct phylogenetic trees based on each of the 11 genes. Comparison of the trees shown in Figure S1 reveals basically concordant topologies and confirms the major divisions I through III. Exception are, in addition to those described for *camp5* and *tly*, the *pak* tree according to which subdivision I-2 is distinct from other division I strains and shares an allele with the majority of division II strains, and three division II strains that cluster among division I strains in the *gms* (strain 18.2.L1) and *zno* trees (strains 36.1.R1 and CCUG50655).

Results of the splits tree analysis, which is able to visualize the contrasting phylogenetic signals in a population affected by recombination, supported the conclusion that recombination, although relatively rare, has contributed to the genetic shaping of the species, particularly evident for clusters I-2, II, and III (Figure S2). This conclusion is supported by statistical significance (*p*<0.0001) in the *phi* test performed in SplitsTree4.

### Clusters and Founders

**Figure 2:**
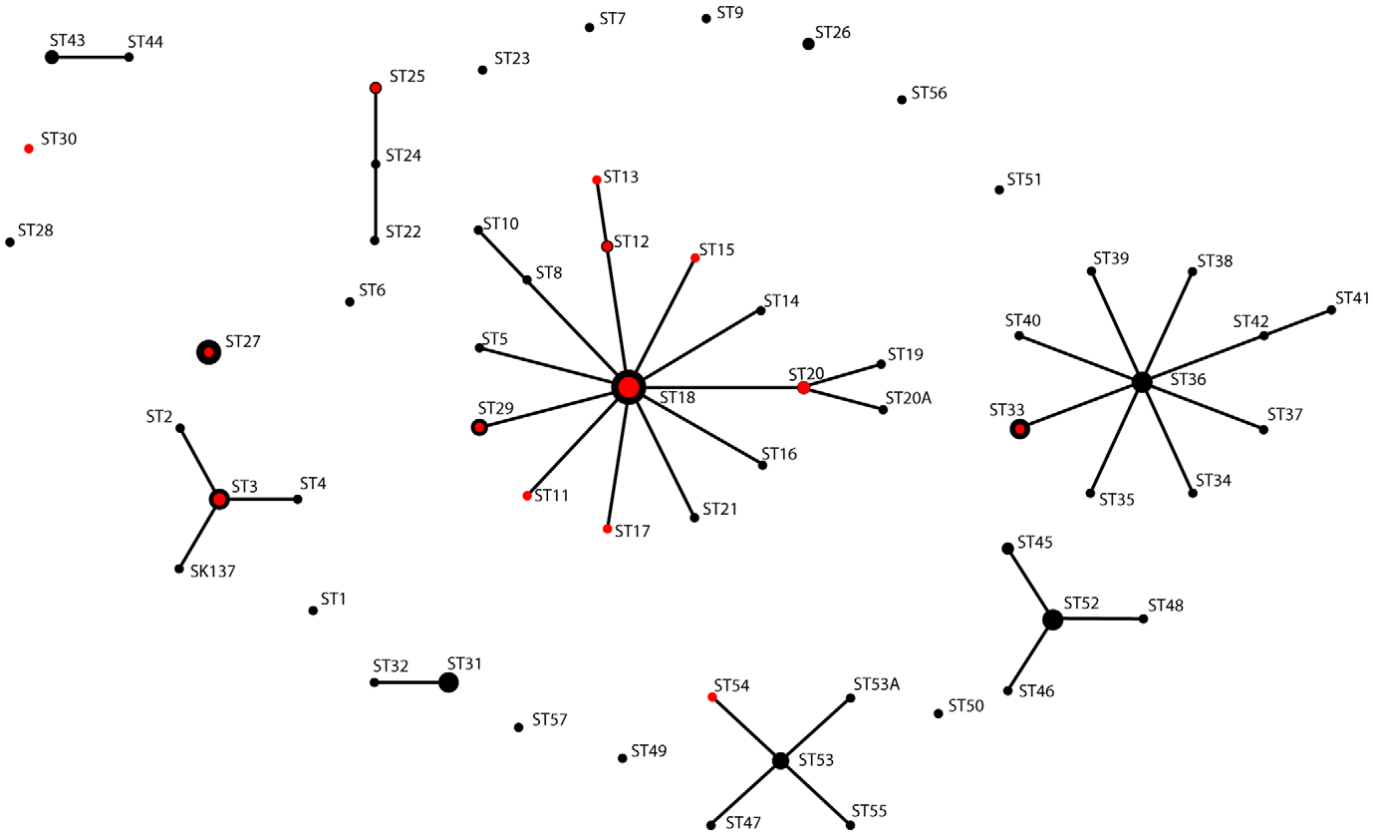
Population snapshot of *P. acnes* identifies a limited number of clone complexes with founders. The eBURST analysis of the allele profile in nine housekeeping and two virulence genes in 143 independent *P. acnes* strains identified eight clusters. Connected members of clusters differed by only one allele out of 11 loci and are assumed to be descended from the same founding genotype. The area of the black circles reflect the number of isolates (see also Figure 1) and the area of the red circles reflect the number of isolates from severe acne. The ST18 cluster included 46 isolates, 14 (30%) of which were from severe acne, whereas the other major cluster, the ST36 cluster, included 23 isolates, one (4.3%) of which was from severe acne. Occasional strains that deviated from the majority allele profile of a certain ST in a single virulence gene are indicated by an A added to the ST number. The founders of the three major ST18, ST36, and ST53 clusters were supported by a bootstrap value of 99, 99, and 78%, respectively.

The eBURST analysis based on allele profiles in the nine housekeeping genes and two virulence genes in the 143 independent isolates identified eight clusters defined as a set of STs and 13 singletons (Figure 2). The founders of the eight clusters, supported by significant boot strap values (78–99%) were identified in an analysis of one representative of each ST. In all cases was the founder the most prevalent among isolates assigned to that cluster.

### Phenotypic Characteristics of *P. acnes* Isolates

In agreement with the species description for *P. acnes*, all isolates were catalase positive, the majority produced indole, and biochemical reactions determined in the API Coryne kit conformed to the identification scheme supplied by the manufacturer. Striking differences were observed in the ability of the 143 non-redundant strains to hydrolyze hyaluronic acid, to express neuraminidase, and to hemolyse horse blood (Table S3). While all strains of divisions I-1 and III lacked hyaluronidase activity, all strains assigned to divisions I-2 and II gave rise to a hydrolysis zone of approximately 1 cm in diameter around the stab growth. Neuraminidase activity was detected in all strains assigned to ST1 through ST30, whereas ST31-57 were negative. Hemolysis was exclusively associated with phylogenetic division I with the exception of all 11 strains of the cluster consisting of STs 1-4, which deviated from other strains of subdivision I-1a also by biotype and *camp5* and *tly* alleles. While most reactions in the API Coryne kit lacked discriminatory power, one interesting exception was the α-glucosidase reaction in which all subdivision I-1 strains were negative except for strains of ST27. The majority of subdivision I-2 strains were positive, division II showed various results, and division III strains were all negative (Table S3).

### Relation to previously reported subdivisions of *P. acnes*

To be able to relate our findings to previously published observations we characterized all 143 independent isolates by biotyping, PFGE typing, and typing based on *recA*, *tly*, and *camp5* sequences. In agreement with the basically clonal population structure, the results correlated, with few exceptions, with the clustering derived from analysis of housekeeping gene sequences (Figure 1). PGFE patterns are shown in Figure S3.

Inclusion of available *recA*, *tly* and *camp5* sequences from previous studies [21], [22] in our phylogenetic analyses revealed correlation between our divisions I, II, and III and divisions previously demonstrated by analysis of sequences of these tree genes with the exception shown in Fig. 1. According to our more detailed phylogenetic analysis, strains designated as subgroup IB in previous reports [21], [22] do not constitute a homogeneous subgroup. Thus, alleles formerly used to define type IB may be found in several distinct lineages, i.e. ST27 and ST28 of cluster I-1a, ST31 and ST32 of cluster 1-1b (biotype 2), and subdivision I-2. Although these strains cluster together according to *recA*, *tly*, *camp5*, and *gms* sequences, they are distinct according to *fba*, *coa*, and *zno* sequences (Figure S1).

### Relation between Phylogenetic Lineages and Origin of Strains

**Figure 3:**
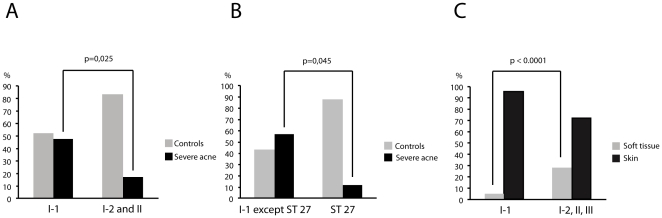
Certain epidemic clones and subpopulations of *P. acnes* are associated with severe acne and soft tissue infections. Percentage of prospectively collected Danish isolates from patients with moderate to severe acne (black bars) versus isolates from healthy controls (grey bars) in A), division I-1 compared to divisions I-2 and II combined; B), ST 27 compared to the rest of division I-1 among Danish isolates; and C), percentage of isolates from blood and infections in normally sterile tissues (see table S4 for details) for division I-1 compared to divisions I-2, II and III among all 143 non-redundant strains included.

Of the 106 non-redundant, prospectively collected Danish strains, 25 were isolated from skin of patients with moderate to severe acne (defined as Leeds score >3), 42 were from mild acne (defined as Leeds score 1-2), and 39 were from healthy controls (Leeds score 0). As indicated by the distribution of Danish moderate to severe acne isolates (Figure 1), 22 such isolates belonged to subdivision I-1, whereas only three isolates clustered in subdivision I-2 and division II and none in division III. Statistical evaluation confirmed a significantly higher proportion of moderate to severe acne isolates in subdivision I-1 as compared to divisions I-2 and II (*p* = 0.025 Fischer's exact test, two-sided) (Figure 3).

One single clone, ST18 in division I-1a, represented 25 independent isolates. These comprised six isolates from severe acne, eight from mild acne, and seven from healthy controls, all living in Denmark and, in addition, three collection strains, i.e. one from blood, Norway 1997, one from acne, London 1920 (NCTC737 = AK25), and one from a wound infection, Sweden 1994. This implies that ST18 is an epidemic clone that has been circulating for at least 87 years in several geographically distinct parts of Europe and frequently is associated with severe acne. The eBURST analysis identified ST18 as the founder of the ST18 complex, which consisted of 46 strains, 14 (30%) of which were from severe acne (Figure 2).

The second largest clone, also part of subdivision I-1, was ST27 comprising 13 isolates. This clone and the single strain in ST28 deviated from other division I-1 by *tly*, *camp5*, and *recA* alleles, by biotype, and by α-glucosidase activity identical to subdivision I-2 strains. Their separate status was supported by the eBURST analysis (Figure 2). Only one of 14 (7.1%) isolates of ST27 and ST28 were from severe acne. Thus, among the prospectively collected Danish isolates, ST27 comprised significantly fewer moderate to severe acne isolates compared to the remaining part of I-1a strains (*p* = 0.045)(Figure 3). The non-acne associated division I-2 constituted the second largest cluster identified by the eBURST analysis, the ST36 cluster (Figure 3). Represented by 23 isolates, only one (4.3%) was from moderate to severe acne.

Statistical analysis of the clustering of all 143 non-redundant isolates showed that those from blood, CSF, and post-surgical hip-prosthesis infections (Figure 1) significantly more often belonged to divisions I-2, II and III than to the acne-associated division I-1 (*p*≤0.0001, Fisher's exact test, two-sided). Details about origin are shown in Table S4.

### Comparison of Genomes

**Figure 4:**
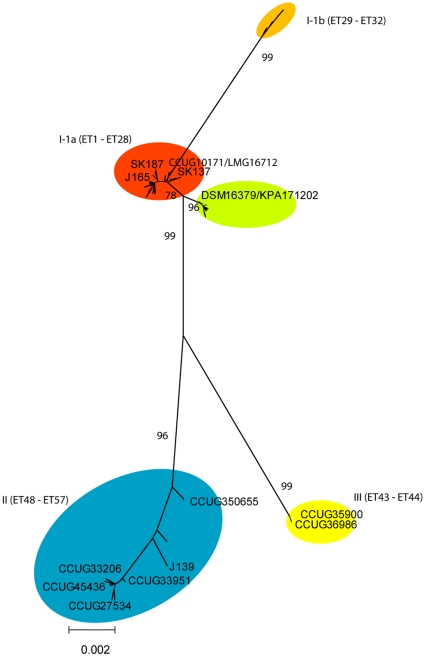
Assignment of genome sequenced strains and relevant collection strains to phylogenetic clusters within *P. acnes*.

To determine the impact of variable genes on the genetic diversification of *P. acnes* and to obtain preliminary information on genes that may distinguish acne- and non-acne associated *P. acnes* strains we compared the complete genomes of strains KPA17202 and SK137. According to the phylogenetic analysis in Figure 1, *P. acnes* strain KPA171202/DSM16379 belongs to division I-2 (ST34), the majority of which is from individuals without acne. Analysis of relevant sequences extracted from the genome of strain SK137 revealed that it belongs in phylogenetic division I-1 (Fig. 4), which is strongly associated with severe acne.

The complete, annotated genome sequence of KPA171202 includes 2,560,265 nucleotides (nt) and that of strain SK137 2,495,334 nt. The corresponding number of identified protein-coding genes (open reading frames) in the two genomes is 2368 and 2352. Alignment of the two genomes showed complete synteny and a high degree of sequence conservation throughout the genomes interrupted by few inserts/deletions. An all-versus-all comparison of the two genomes using BlastP with cut-off values ≥70% identity and ≥70% coverage was performed. The comparison identified 124 genes unique to strain KPA171202, of which 53 encode hypothetical proteins, and 62 genes unique to strain SK137, of which 39 encode hypothetical proteins.

Each protein identified as being unique to KPA171202 and SK137, respectively, in the initial pairwise comparison was blasted against three non-closed *P. acnes* genomes, which according to our phylogenetic analysis represent divisons I-1a (J165 and SK187) and II (J139) (Fig. 4). The results of the genome comparisons are summarized in Table S5 and show both strain-specific and cluster-specific genes and gene complexes. Of the variably present genes, a total of 106 constituted five of 11 island-like regions identified with atypical codon usage in the KPA171202 genome by Brüggemann et al. [25]. One of these (KPA171202 loci PPA1596, 1583–1613) is a prophage and was only present in KPA171202. Another (PPA846–874 and 2354, 2400, and 2355) contained several transposases and a conjugal transfer system and was present only in KPA171202 and SK187. The third KPA171202 genome island (PPA1278–1304) included a plasmid partition protein in addition to genes involved in biosurfactant production and was present in all genomes apart from SK137. The two remaining islands (Table S5) did not include genes usually associated with mobile elements.

Strikingly, all three genomes of strains assigned to phylogenetic division I-1a (SK137, J165, SK187) lacked a total of 10 genes associated with carbohydrate metabolism (glycosidases, permeases and kinases) in KPA171202 and J139, including hyaluronidase in agreement with our phenotypic analysis ( Tables S3 and S5).

Genes unique to SK137 and the two other representatives of the acne-associated division I-1a included one cluster (HMPREF0675_4518–4528) and two singletons (4648 and 4712) encoding hypothetical proteins. The cluster contains two glycosyl transferases that belong to the chitinase superfamily of proteins.

## Discussion

Acne is the most frequent dermatologic disease, often appearing at puberty at a vulnerable point in life where the patients are striking to find their role as grown ups. At this age the sebaceous glands enlarge due to hormonal influences and the number of *P. acnes* rises from an almost undetectable level. *P. acnes* not only becomes one of the dominant and ubiquitous members of the commensal skin microbiota and an exclusive inhabitant of healthy sebaceous follicles, but is also suspected to induce the inflammatory papules and pustules of acne, and may even provoke the initial hyper-keratinisation of the sebaceous duct that starts the acne process [1], [3], [9], [26], [27]. Based on analysis of 210 *P. acnes* isolates we tested the hypothesis that these apparently contradictory host relationships are due to certain evolutionary lineages of *P. acnes* being virulent and associated with acne while others are compatible with health. The results of our study support this hypothesis and further suggests that strains that are able to cause infections in normally sterile tissues and body fluids belong to the parts of the *P. acnes* population that are commensals of healthy skin.

Analysis of partial sequences of nine housekeeping genes evenly distributed along the *P. acnes* genome revealed a sequence divergence (0.01) identical to that of the other major member of the skin microbiota, *Staphylococcus epidermidis* [28]. This level is remarkably lower than the genetic distances observed within species of commensal streptococci of the upper respiratory tract (*Streptococcus oralis*, 0.097; *Streptococcus mitis*, 0.05) [29] but similar to that of selected bacterial mucosal pathogens such as *Streptococcus pneumoniae* (0.01), and *Haemophilus influenzae* (0.02)(distances calculated in MEGA4 on the basis of concatenated sequences downloaded from www.mlst.net). The observed close similarity or identity of Chinese and Scandinavian isolates suggest that adding more strains from diverse locations and disease is unlikely to result in significantly increased overall genetic diversity.

Although multilocus sequence typing, usually based on seven gene loci [30], [31], but in this study nine loci, is a well-established typing method with high discriminatory power, only 57 sequence types (STs) were detected among the examined 210 isolates, and single STs were represented by up to 25 independent isolates. The eBURST analysis even suggests that a major part of these STs are recent descendents from a few founders (Figure 2). This shows an unexpected sharing of clones of *P. acnes* in the human population in sharp contrast to observations for members of the commensal microbiota of the gastrointestinal and upper respiratory tracts [32], [33]. The finding of single clones represented by up to 25 isolates from diverse geographic areas collected with 87 years interval indicates relative genetic stability and shows that some epidemic clones are capable of successfully spreading in the human population. This is compatible with the previous observation that cross-infection with *P. acnes* strains readily occurs in dermatological clinics [34] and, thus, presumably wherever direct or indirect human contact is occurring. Further studies are needed to evaluate if this ability is restricted to certain clones, as suggested by our findings, and to determine to what extend such clones become permanent members of the skin microbiota or are transient colonizers.

Analysis of genes encoding two surface-exposed putative virulence factors, the CAMP 5 protein (*camp5*) and the putative cytotoxin/hemolysin (*tly*), revealed the same sequence polymorphism as observed in housekeeping genes (Table S1). This indicates a lack of selection for enhanced diversification as usually observed for pathogens infecting mucosal membranes [35] presumably reflecting a limited impact of adaptive immunity on bacteria that colonize the surface of skin. In this context it is interesting that strains of division II, which include a significantly higher proportion of isolates from blood and soft tissue infections, showed considerably more polymorphism in *camp5* and *tly* genes (Figure 1).

Bacteria may undergo genetic diversification by spontaneous accumulation of mutations, as reflected in the alleles of housekeeping genes detected by MLST, by homologous recombination affecting genes shared by the involved clones, and by horizontal transfer of genes variably present in the population. The overall similarity, but not identity, of the topography of the trees based on individual housekeeping genes (Figure S1), the clear linkage disequilibrium observed for genetic and phenotypic characteristics (Figure 1 and Table S3), and the results of the Splits tree analysis (Figure S2) show that the population structure of *P. acnes* is basically clonal, yet, to some extend, affected by homologous recombination. One example illustrating the latter is the *recA*, *camp5*, *gms*, and *tly* sequences of part of the I-1 population (ST27, ST28, ST31, and ST32), which clearly were related to corresponding sequences of strains of the I-2 population (Figure S1). The fact that all these genes are located within one section of the genome spanning close to 420,000 nt suggest conjugal replacement of large genome segments similar to what was reported recently for *Streptococcus agalactiae* [36].

The limited diversity detected in the core genome and the complete synteny, was also reflected in a limited number of variably present genes accounting for only 2–5% of the total number of genes in the two complete genomes that represent divisions I-1 and I-2. This is in striking contrast to observations made for several other bacterial species [18], [37]. Many of these variably present genes are located in clusters that, by their aberrant codon usage, suggest recent import from other bacteria [25]. This suggests that *P. acnes*, although characterized by a highly conserved core genome, has the potential for ecological flexibility as indicated by the striking loss of genes involved in carbohydrate metabolism in division I-1 strains relative to the division I-2 and division II strains. The compensatory gain of neuraminidase and chitinase activity in division I-1 strains may allow them to cleave host glycoproteins and cell wall chitin of the fungus *Malassezia*, which co-inhabits sebaceous follicles. Further studies are required to substantiate this and to evaluate its potential clinical implications.

The three major genetic divisions I to III identified by the phylogenetic analysis of concatenated sequences of nine housekeeping genes correspond to a large degree to those previously defined by analysis of *recA*, *tly* and *camp5* sequences [21], [22], [38]. Because of clear discrepancies in the genetic structure of subdivisions, we propose a new nomenclature for the phylogenetic clusters as shown in Figure 1. Although biotypes largely correlated with the genetic clusters we propose that MLSA is the preferred typing method due to its superior reproducibility, the portable nature of the results, and the ability to compare data from different laboratories.

To our knowledge this is the first study to demonstrate that a distinct subpopulation of *P. acnes* is significantly associated with moderate to severe acne as defined by the Leeds acne score. As our analysis of the initial strain collection, which included up to six strains from single individuals, demonstrated that individuals may carry several different clones, all isolates from acne patients may not necessarily be involved in the pathogenesis of the condition. The statistical significance of the association of isolates from division I-1 with moderate to severe acne (*p* = 0.025) observed in spite of this weakness emphasizes its validity (Figure 3). Within division I-1a 30% of isolates of the epidemic ST18 and its descendents in the ST18 complex were from severe acne patients suggestive of particular virulence and dissemination potential.

The crucial question is which properties explain the different association with disease. Indeed, previous *in vitro* studies show significant differences that, with few reservations, can be translated to our phylogenetic clusters. Thus, division I-1 strains are the most potent inducers of inflammation and stimulation of sebocytes and keratinocytes, the latter by selective induction of human β-defensin-2 and interleukin-8 through Toll-like receptors 2 and 4 [5], [6]. However, the exact constituents of *P. acnes* that may explain such differences are not clear. Our phenotypic analyses and comparisons of five representative genomes reveal differences, of which some support and others are contrasting with previous conclusions. Our results confirm differences in neuraminidase expression [39] and support the assumption that this activity may be a virulence factor [25], [40] as it was exclusively associated with the acne-associated division I-1. The genetic basis of this difference is not clear as all five representative genomes included three highly conserved genes annotated as sialidases. In contrast, strains of divisions associated with acne lacked hyaluronidase activity and the corresponding gene previously assumed to be a virulence factor in acne [25]. Likewise, a previous study demonstrated more abundant CAMP factor release by strains of divisions I-2 and II which, according to our findings, are not associated with acne [21].

Lipases of *P. acnes* are thought to play an important role in the pathogenesis of acne, as release of irritant fatty acids may contribute to inflammation [41]. In a study of several strains also included in our study Coenye et al. [41] demonstrated a significant overproduction of oleate-degrading lipase activity in biofilm-grown ST18 strain NCTC737, compared to ST27 strain CCUG10171, and in particular ST57 strain CCUG33206 (Fig. 4). Their most potent strain belongs to ST18, the acne-associated epidemic clone of division I-1, while ST27 is the exceptional non-acne associated division I-1 clone with aberrant biotype, *camp5* and *tly* allele, and α-glucosidase activity. The weakest activity was observed in ST57 belonging to our division II, which is unrelated to acne.

The results of our study provide a convincing explanation to the long-term controversy on the significance of *P. acnes* in relation to acne. While some evolutionary lineages of the species are associated with health, others, including an epidemic clone, are strongly associated with moderate to severe acne. The fact that this dichotomy correlates with previously observed differences in the *in vitro* expression of putative virulence factors strongly support the conclusion that acne is an infectious disease affecting genetically susceptible individuals. The study provides support for certain hypothetical virulence factors while rejecting others. Comparative genome- and function-based studies of thoroughly characterized disease- and non-disease associated strains may identify targets for effective treatment and prevention of acne.

## Materials and Methods

### Ethics statement

The study protocol was approved by the Ethics Committee of the County of Aarhus, and the study was conducted according to the principles of the declaration of Helsinki. Written informed consent was obtained from study participants and/or their legal guardians.

### Bacterial isolates

The 210 isolates of *P. acnes* included in the study comprised 105 *P. acnes* isolates from 25 acne patients and 55 isolates from 14 controls without skin disease, all Caucasians recruited in Aarhus, Denmark. All subjects were clinically examined by the same specialist in dermatology (HBL) and acne was graded according to the Leeds score [42]. To achieve a broader geographic and spatial representation we included 20 isolates collected in 2008 from five healthy Chinese individuals living in Beijing, China, and 30 strains from recognized public collections representing clinical isolates from the United Kingdom, U.S.A., Sweden, Norway, and Germany collected between 1920 to 2004 mainly from opportunistic infections (Table S4).

### Skin samples and isolation of *P. acnes*

From all the Danish subjects, separate samples were obtained from the surface of the skin on the face and the upper part of the back by the detergent scrub technique of Williamson and Kligman [43], in patients from acne-affected areas. In addition, sebaceous follicles were sampled by cyanoacrylate biopsy [44] from a facial area with acne and in controls from the side of the nose. The follicular casts were dissected with a scalpel under a stereo microscope. Four to 15 casts were dissolved in 1.5 ml of wash fluid with 0.2g of Ballatoni beads and mixed for 1 minute on a Whirly mixer [45]. Samples from the Chinese healthy controls were taken from the face. The samples were inoculated on TYEG agar supplemented with 2 µg/ml of furazolidone [34] and on 5% blood agar incubated under anaerobic conditions for 96 hours. From cultures of each sample a single colony of *P. acnes* was randomly chosen for inclusion in the study after verification of its identity.

### Phenotypic analysis and biotyping

All isolates were examined by Gram staining, tested for production of indole and for catalase activity using standard methodology. At least one representative of each sequence type was examined in the API Coryne kit (bioMérieux, Marcy-lÉtoile, France), which includes 20 biochemical tests. Their identification as *P. acnes* was further confirmed by demonstrating homology of the 16S rRNA gene sequence with that of the designated type strain of the species using primers previously reported [29].

All strains were assigned to one of five biotypes by a modification of the method described by Kishishita et al. [23]. In brief, the strains were inoculated in basal medium (trypticase 1g/dl, yeast extract 0.3 g/dl, heart extract 0,3 g/dl, NaCl 0.2 g/dl, L-cysteine-HCl 0.03 g/dl, agar 0.1 g/dl, Tween 80 0.025 ml/dl, pH 7.0) and in basal medium supplemented with 1g /dl of each of the carbohydrates ribose, sorbitol, erythritol, and glucose (positive control). After incubation for seven days at 37° C the pH of each culture and of un-inoculated control media with and without the respective sugars was measured. For each sugar the final pH values was measured and the distribution of measurements was recorded. For all four sugars a bimodal distribution was observed and positive, indeterminate, and negative reactions were defined accordingly. Un-inoculated media showed a pH of 6.5–6.9 (ribose 6.1–6.7). Positive reactions were defined as pH≤4.99, indeterminate reactions as pH 5.0–6.49, and negative reactions as pH≥6.50. Each strain was assigned to biotypes 1 to 5 (B1–5) defined by the ability to ferment ribose, erythritol and sorbitol as follows: B1: + + +, B2: + + −, B3: + − +, B4: + − −, and B5: − − − according to Kishishita et al. [23].

Hemolysis was recorded on 5% horse blood agar (Statens Serum Institut, Copenhagen, Denmark) following anaerobic incubation for 7 days. Hyaluronidase activity was demonstrated by incubating the strains for four days under anaerobic conditions as stab cultures on agar plates containing hyaluronic acid as described [46].

### PCR and sequencing of bacterial DNA

For preparation of bacterial DNA a loopfull of bacteria was suspended in 100 µl of double sterilized water. Twenty µl of this solution was mixed with 80 µl 0.05 M NaOH and incubated at 60°C for 45 minutes. Subsequently, 9.2 µl of 1M Tris-HCl, pH 7.0 were added and the solution was diluted 1∶100. Five µl of this solution was used for PCR. Internal fragments of the housekeeping genes *cel* (Transcription regulator CelR), *coa* (O-succinylbenzoate-CoA synthase), *fba* (Fructose bisphosphate aldolase), *gms* (Glutamyl-tRNA synthetase), *lac* (L-lactate dehydrogenase), *oxc* (Cytochrome c oxidase subunit II), *pak* (Pantothenate kinase), *recA*, *zno* (Zn-dependant alcohol dehydrogenase) were amplified by PCR and sequenced using primers listed in Table S6. These gene loci and the primers were recommended for analysis of *P. acnes* at the MLST project internet site (www.mlst.net) except for *recA*, which we substituted for *cob* (Cobalamin biosynthesis CobD/CbiB protein), to allow reference to previously reported genotypes of the species. In addition, two putative virulence factor genes *tly* (putative cytotoxin/hemolysin) and *camp5* (co-haemolytic CAMP factor 5) were amplified as described [21], [22] (Table S4). Primers were purchased from DNA Technology (Aarhus, Denmark). For the PCR we used approximately 1 ng whole-cell DNA as template and either Ready-To-Go PCR beads (Amersham Pharmacia Biotech) or Eppendorf (5prime) hotmastermix (Eppendorf Nordic). The temperature profile for the PCR was an initial denaturation at 96°C for 40 s, followed by 35 cycles at 94°C for 35 s, 55°C for 40 s, and 72°C for 40 s, followed by a final extension at 72°C for 7 min. Amplicons were purified using Wizard Minicolumns (Promega, Madison, Wis.). Sequencing of both strands of the amplified fragments was achieved with the same primers and the Thermo Sequenase dye terminator cycle sequencing kit (Amersham Life Science) on Applied Biosystems automated sequencers 3730xl and PRISM 377 (Perkin-Elmer Applied Biosystems).

### Phylogenetic analysis

Phylogenetic and molecular evolutionary analyses were conducted using MEGA version 4.0 software [47]. The Minimum evolution algorithm was applied based on the Nucleotide Maximum Composite Likelihood analysis of all positions. Bootstrap analysis was based on 500 replicates. For comparison, analyses were also conducted with the Neighbor Joining algorithm. For the *recA*, *tly*, and *camp5* genes, sequences from previous studies deposited in public sequence databases were included in the analyses to allow reference to such studies. Estimates of genetic divergence at each gene locus were obtained in MEGA 4.0 and SplitsTree4 [48] was used to compute unrooted phylogenetic networks from the concatenated sequences and for analysis of evidence of genetic recombination in the *P.acnes* population. The eBURSTv3 programme (http://eburst.mlst.net/default.asp) was used to identify clonal complexes and founders of such complexes according to Feil et al. [49].

### Pulsed-field gel electrophoresis

Pulsed-field gel electrophoresis (PFGE) analysis of genomic DNA was performed as described by Oprica et al. [24] using the restriction endonuclease *Spe*-I. Comparison of PFGE patterns was done by visual analysis.

### In silico whole genome comparison

The complete, annotated genome sequences of *P. acnes* strain KPA171202/DSM16379 (NC_006085) and strain SK137 (NC_014039) were compared. The origin of KPA171202/DSM16379 is referred to as “contamination of anaerobic culture” (Deutsche Sammlung von Mikroorganismen und Zellkulturen Catalogue) and SK137 as isolated from human skin. In addition, the non-closed genomes of strains SK187 (NZ_ADJM00000000), J165 (NZ_ADJL00000000), and J139 (NZ_ADFS00000000) were included in the analysis. The four latter genomes serve as reference for the Human Microbiome Project. The two complete genomes were aligned using MAUVE [50] and ClustalX. To identify strain-specific gene pairs of orthologous genes, bidirectional BLASTp comparisons using the cut-off values ≥70% identity and ≥70% coverage. All protein sequences identified by this analysis were blasted against the remaining genomes.

#### Statistical analysis

Statistical significance was tested with Fisher's exact test, two-sided, based on 2×2 tables in Stata software (StataCorp, TX).

## Supporting Information

Figure S1 Basically concordant phylogenetic trees based on sequences of individual genes encoding 9 housekeeping proteins and 2 putative virulence factors are evidence of limited recombination.
(0.33 MB PDF)

Figure S2 Evidence of limited recombination in the *P. acnes* population.
(0.18 MB PDF)

Figure S3 Limited PFGE pattern polymorphism in *P. acnes*.
(0.50 MB PDF)

Table S1 Genetic diversity in 9 housekeeping and 2 virulence genes of *P. acnes*.
(0.04 MB DOC)

Table S2 Allele profiles of the 57 STs in the nine housekeeping and two virulence genes.
(0.13 MB DOC)

Table S3 Phenotypic characteristics of *P. acnes* isolates assigned to each phylogenetic division.
(0.03 MB DOC)

Table S4 *P. acnes* strains from public collection included in the study and their assignment to genetic divisions and sequence type (ST).
(0.06 MB DOC)

Table S5 Genes identified as missing in one of the complete genomes of *P. acnes* strains KPA171202 and SK137 and their presence in the genomes of three additional genomes representing distinct evolutionary clusters.
(0.07 MB DOC)

Table S6 Primers used for amplification and sequencing of *P. acnes* gene loci.
(0.04 MB DOC)
