# Supplementary material for: Population Genetic Analysis of Propionibacterium acnes Identifies a Subpopulation and Epidemic Clones Associated with Acne
Source: PLoS One. 2010 Aug 19;5(8):e12277. doi: 10.1371/journal.pone.0012277 (PMC2924382; doi:10.1371/journal.pone.0012277)
Supplement: Figure S1 — Basically concordant phylogenetic trees based on sequences of individual genes encoding 9 housekeeping proteins and 2 putative virulence factors are evidence of limited recombination. (0.33 MB PDF) [file pone.0012277.s001.pdf]

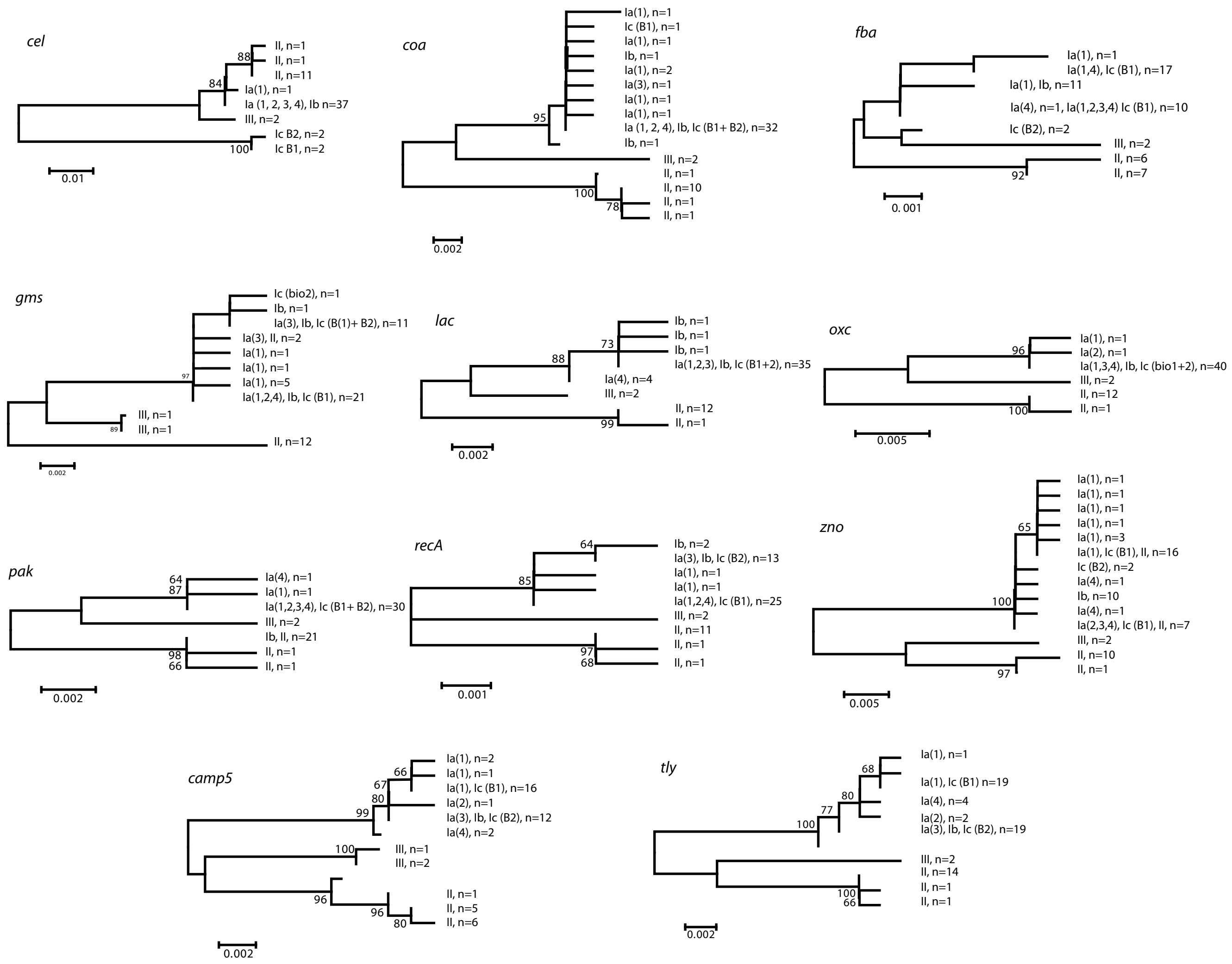

Figure S1. Basically concordant phylogenetic trees based on sequences of individual genes encoding 9 housekeeping proteins and 2 putative virulence factors are evidence of limited recombination. The trees were constructed by the Minimum evolution principle. Comparison of the trees illustrates how the topology of the tree based on *cel* deviated significantly from other trees in separating strains of the four STs of cluster I-1b from all other strains in the collection. With few exceptions, the overall topologies of the trees based on the remaining 10 genes are concordant and confirm the separation of the major divisions I, II, and III indicative of limited recombination between these divisions.
An exception is the *pak* tree according to which subdivision I-2 is distinct from other division I strains and shares an allele with the majority of division II strains. Likewise, three division II strains cluster among division I strains in the *gms* (strain 18.2.L1) and *zno* trees (strains 36.1.R1 and CCUG50655) providing further evidence of recombination, though limited in extent.
