# Supplementary material for: Population Genetic Analysis of Propionibacterium acnes Identifies a Subpopulation and Epidemic Clones Associated with Acne
Source: PLoS One. 2010 Aug 19;5(8):e12277. doi: 10.1371/journal.pone.0012277 (PMC2924382; doi:10.1371/journal.pone.0012277)
Supplement: Figure S2 — Evidence of limited recombination in the P. acnes population. (0.18 MB PDF) [file pone.0012277.s002.pdf]

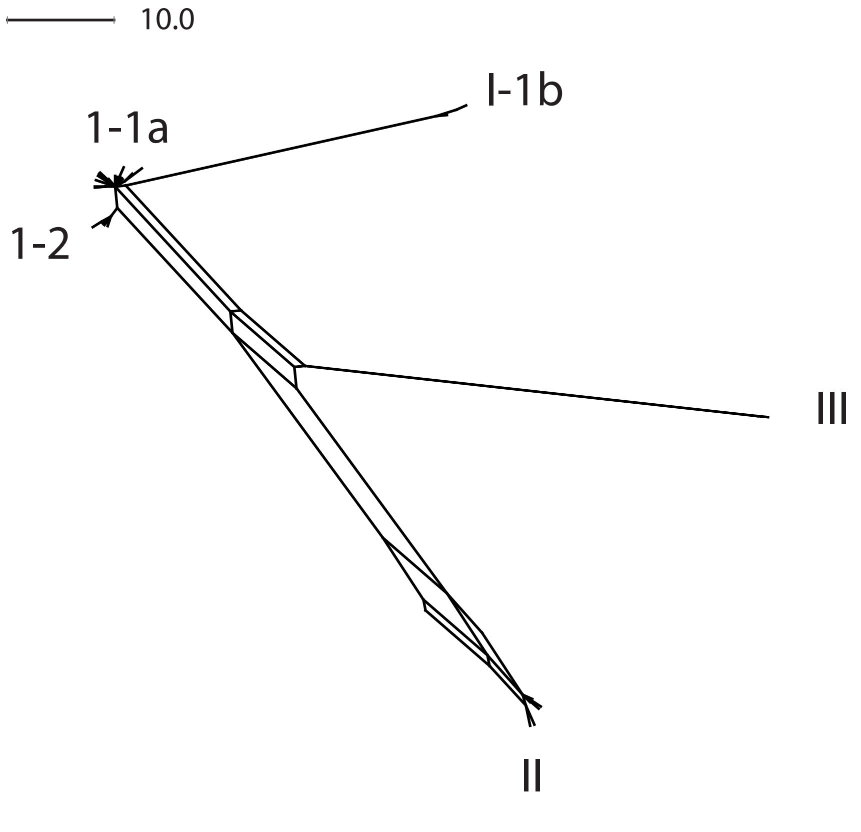

Figure S2. Evidence of limited recombination in the *P. acnes* population. Parsimonious Splits tree based on concatenated sequences of 9 housekeeping gene loci performed with the SplitsTree4 software. The tree shows evidence of recombination playing a role in shaping the genomes of clusters Ib, II, and III.
