# Supplementary material for: Population Genetic Analysis of Propionibacterium acnes Identifies a Subpopulation and Epidemic Clones Associated with Acne
Source: PLoS One. 2010 Aug 19;5(8):e12277. doi: 10.1371/journal.pone.0012277 (PMC2924382; doi:10.1371/journal.pone.0012277)
Supplement: Figure S3 — Limited PFGE pattern polymorphism in P. acnes. (0.50 MB PDF) [file pone.0012277.s003.pdf]

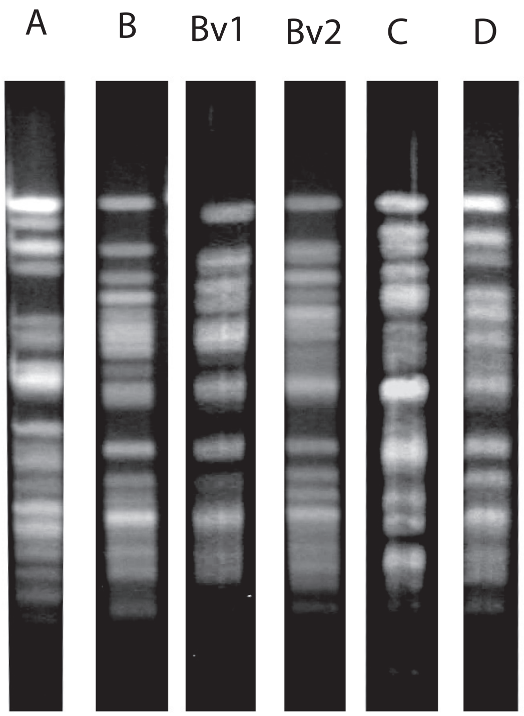

Figure S3. Limited PFGE pattern polymorphism in *P. acnes*. Four patterns (A –D) observed after pulsed-field analysis of *P. acnes* genomic DNA digested with the restriction endonuclease Spe-I. Two minor variants of pattern B are shown (Bv1 and Bv2).
