# Supplementary material for: Population Genetic Analysis of Propionibacterium acnes Identifies a Subpopulation and Epidemic Clones Associated with Acne
Source: PLoS One. 2010 Aug 19;5(8):e12277. doi: 10.1371/journal.pone.0012277 (PMC2924382; doi:10.1371/journal.pone.0012277)
Supplement: Table S1 — Genetic diversity in 9 housekeeping and 2 virulence genes of P. acnes. (0.04 MB DOC) [file pone.0012277.s004.doc]

**Table S1. Genetic diversity in 9 housekeeping and 2 virulence genes of *P. acnes.***

| Locus | Fragment size (bp)^1^ | No. of  alleles | No. of polymorphic/  informative sites | % polymorphic sites | Mean genetic distance ± SE |
| --- | --- | --- | --- | --- | --- |
| *camp5* | 834 | 12 | 33/27 | 3.96 | 0.011 ± 0.002 |
| *cel* | 363 | 8 | 42/39 | 11.57 | 0.017 ± 0.003 |
| *coa* | 528 | 15 | 32/22 | 6.06 | 0.012 ± 0.003 |
| *fba* | 513 | 8 | 10/9 | 1.95 | 0.005 ± 0.002 |
| *gms* | 474/462 | 11 | 22/17 | 4.64 | 0.011 ± 0.003 |
| *lac* | 420 | 8 | 14/10 | 3.33 | 0.008 ± 0.002 |
| *oxc* | 372 | 6 | 17/14 | 4.57 | 0.011 ± 0.003 |
| *pak* | 393 | 7 | 11/7 | 2.80 | 0.007 ± 0.003 |
| *recA* | 807 | 9 | 15/11 | 1.86 | 0.003 ± 0.001 |
| *tly* | 777 | 11 | 36/31 | 4.63 | 0.012 ± 0.002 |
| *zno* | 417 | 14 | 35/29 | 8.39 | 0.021 ± 0.004 |

^1^ Three independent isolates, two from Sweden (CCUG35900 and CCUG35986) and one from Norway (CCUG35749), had an in-frame deletion of 12 nucleotides in the *gms* gene (positions 752-763 in the complete gene). Strain CCUG35547, a Swedish isolate otherwise identical to the three, did not have this deletion in *gms*.
