# Supplementary material for: Population Genetic Analysis of Propionibacterium acnes Identifies a Subpopulation and Epidemic Clones Associated with Acne
Source: PLoS One. 2010 Aug 19;5(8):e12277. doi: 10.1371/journal.pone.0012277 (PMC2924382; doi:10.1371/journal.pone.0012277)
Supplement: Table S2 — Allele profiles of the 57 STs in the nine housekeeping and two virulence genes. (0.13 MB DOC) [file pone.0012277.s005.doc]

**Table S2. Allele profiles of the 57 STs in the nine housekeeping and two virulence genes.^1^**

| **ST** | **Isolate** | ***cel*** | ***coa*** | ***fba*** | ***gms*** | ***lac*** | ***oxc*** | ***pak*** | ***recA*** | ***zno*** | ***camp5*** | ***tly*** |
| --- | --- | --- | --- | --- | --- | --- | --- | --- | --- | --- | --- | --- |
| 1 | 42.1.R1 | 5 | 9 | 4 | 8 | 5 | 3 | 1 | 5 | 8 | 6 | 5 |
| 2 | 37.1.R1 | 5 | 9 | 4 | 8 | 5 | 3 | 3 | 5 | 10 | 6 | 5 |
| 3 | 3.1.A1 | 5 | 9 | 4 | 8 | 5 | 3 | 3 | 5 | 11 | 6 | 5 |
| 4 | 19.1.L1 | 5 | 9 | 2 | 8 | 5 | 3 | 3 | 5 | 11 | 6 | 5 |
| 5 | 14.1.L1 | 5 | 1 | 2 | 8 | 4 | 3 | 3 | 5 | 6 | 3 | 3 |
| 6 | CCUG50480 | 5 | 3 | 2 | 8 | 4 | 3 | 3 | 5 | 2 | 3 | 3 |
| 7 | 40.1.R1 | 5 | 9 | 2 | 8 | 4 | 1 | 3 | 4 | 6 | 3 | 3 |
| 8 | China 7.1 | 5 | 9 | 2 | 8 | 4 | 3 | 3 | 5 | 5 | 3 | 3 |
| 9 | China 4.1 | 5 | 6 | 2 | 8 | 4 | 3 | 2 | 5 | 5 | 3 | 3 |
| 10 | China 8.1 | 5 | 9 | 2 | 4 | 4 | 3 | 3 | 5 | 5 | 3 | 3 |
| 11 | 34.2.R1 | 5 | 9 | 1 | 8 | 4 | 3 | 3 | 5 | 6 | 3 | 3 |
| 12 | 33.1.A1 | 5 | 5 | 2 | 8 | 4 | 3 | 3 | 5 | 6 | 3 | 3 |
| 13 | 37.1.L1 | 4 | 5 | 2 | 8 | 4 | 3 | 3 | 5 | 6 | 3 | 3 |
| 14 | 40.1.L1 | 5 | 9 | 2 | 8 | 4 | 3 | 3 | 5 | 3 | 3 | 3 |
| 15 | 20.2.A1 | 5 | 9 | 2 | 8 | 4 | 3 | 3 | 5 | 4 | 3 | 3 |
| 16 | 21.1.A1 | 5 | 7 | 2 | 8 | 4 | 3 | 3 | 5 | 6 | 3 | 3 |
| 17 | 26.2.A1 | 5 | 9 | 2 | 5 | 4 | 3 | 3 | 5 | 6 | 3 | 3 |
| 18 | 1.4.L1 | 5 | 9 | 2 | 8 | 4 | 3 | 3 | 5 | 6 | 3 | 3 |
| 19 | CCUG34938 | 5 | 9 | 2 | 7 | 4 | 3 | 3 | 5 | 1 | 3 | 3 |
| 20 | 12.1.R1 | 5 | 9 | 2 | 7 | 4 | 3 | 3 | 5 | 6 | 3 | 3 |
| 20A | CCUG38584 | 5 | 9 | 2 | 7 | 4 | 3 | 3 | 5 | 6 | 3 | 1 |
| 21 | 19.1.R1 | 5 | 9 | 4 | 8 | 4 | 3 | 3 | 5 | 6 | 3 | 3 |
| 22 | 4.4.L1 | 5 | 9 | 4 | 7 | 4 | 3 | 3 | 5 | 6 | 1 | 3 |
| 23 | 18.1.R1 | 5 | 9 | 4 | 7 | 4 | 3 | 3 | 3 | 6 | 3 | 3 |
| 24 | 4.4.R1 | 5 | 9 | 3 | 7 | 4 | 3 | 3 | 5 | 6 | 1 | 3 |
| 25 | 23.1.L1 | 5 | 9 | 3 | 8 | 4 | 3 | 3 | 5 | 6 | 1 | 3 |
| 26 | China 2.1 | 5 | 9 | 4 | 8 | 4 | 2 | 3 | 5 | 11 | 4 | 4 |
| 27 | 1.5.L1 | 5 | 9 | 4 | 6 | 4 | 3 | 3 | 2 | 11 | 5 | 6 |
| 28 | 25.1.R1 | 5 | 8 | 4 | 3 | 4 | 3 | 3 | 2 | 11 | 5 | 6 |
| 29 | 27.1.R1 | 7 | 9 | 2 | 8 | 4 | 3 | 3 | 5 | 6 | 3 | 3 |
| 30 | 20.2.R1 | 7 | 2 | 4 | 3 | 4 | 3 | 3 | 2 | 11 | 5 | 6 |
| 31 | 3.6.A1 | 8 | 9 | 5 | 3 | 4 | 3 | 3 | 2 | 7 | 5 | 6 |
| 32 | 16.2.R1 | 8 | 9 | 5 | 1 | 4 | 3 | 3 | 2 | 7 | 5 | 6 |
| 33 | CCUG32901 | 5 | 9 | 4 | 3 | 4 | 3 | 5 | 2 | 9 | 5 | 6 |
| 34 | DSM16379 | 5 | 9 | 3 | 8 | 4 | 3 | 5 | 2 | 9 | 5 | 6 |
| 35 | 2.3.A1 | 5 | 10 | 3 | 3 | 4 | 3 | 5 | 2 | 9 | 5 | 6 |
| 36 | 21.1.L1 | 5 | 9 | 3 | 3 | 4 | 3 | 5 | 2 | 9 | 5 | 6 |
| 37 | 21.2.A1 | 5 | 9 | 3 | 2 | 4 | 3 | 5 | 2 | 9 | 5 | 6 |
| 38 | 27.1.A1 | 5 | 9 | 3 | 3 | 2 | 3 | 5 | 2 | 9 | 5 | 6 |
| 39 | 18.2.A1 | 5 | 9 | 3 | 3 | 1 | 3 | 5 | 2 | 9 | 5 | 6 |
| 40 | 27.1.L1 | 5 | 9 | 3 | 3 | 3 | 3 | 5 | 2 | 9 | 5 | 6 |
| 41 | 36.1.L1 | 5 | 4 | 3 | 3 | 4 | 3 | 5 | 1 | 9 | 5 | 6 |
| 42 | CCUG36661 | 5 | 9 | 3 | 3 | 4 | 3 | 5 | 1 | 9 | 5 | 6 |
| 43 | CCUG35900 | 6 | 11 | 6 | 9 | 6 | 4 | 4 | 9 | 12 | 8 | 8 |
| 44 | CCUG35547 | 6 | 11 | 6 | 10 | 6 | 4 | 4 | 9 | 12 | 8 | 8 |
| 45 | 36.1.R1 | 3 | 13 | 8 | 11 | 7 | 5 | 5 | 6 | 11 | 11 | 9 |
| 46 | CCUG50655 | 3 | 13 | 8 | 11 | 7 | 5 | 5 | 6 | 6 | 11 | 9 |
| 47 | 18.2.L1 | 3 | 13 | 7 | 6 | 7 | 5 | 5 | 6 | 14 | 10 | 9 |
| 48 | CCUG33951 | 3 | 13 | 8 | 11 | 7 | 5 | 5 | 6 | 13 | 11 | 9 |
| 49 | China 2.3 | 3 | 12 | 7 | 11 | 7 | 5 | 5 | 6 | 14 | 9 | 9 |
| 50 | 7.1.L1 | 1 | 14 | 8 | 11 | 7 | 5 | 5 | 6 | 14 | 11 | 9 |
| 51 | CCUG27534 | 3 | 13 | 8 | 11 | 7 | 6 | 7 | 6 | 14 | 12 | 10 |
| 52 | 5.1.R1 | 3 | 13 | 8 | 11 | 7 | 5 | 5 | 6 | 14 | 11 | 9 |
| 53 | 18.1.A1 | 3 | 13 | 7 | 11 | 7 | 5 | 5 | 6 | 14 | 10 | 9 |
| 53A | CCUG36609 | 3 | 13 | 7 | 11 | 7 | 5 | 5 | 6 | 14 | 10 | 11 |
| 54 | 34.1.A1 | 3 | 13 | 7 | 11 | 7 | 5 | 5 | 7 | 14 | 10 | 9 |
| 55 | CCUG45436 | 3 | 13 | 7 | 11 | 8 | 5 | 5 | 6 | 14 | 10 | 9 |
| 56 | 39.3.R1 | 3 | 13 | 7 | 11 | 7 | 5 | 6 | 8 | 14 | 11 | 9 |
| 57 | CCUG33206 | 2 | 15 | 7 | 11 | 7 | 5 | 5 | 6 | 14 | 10 | 9 |

^1^ STs were defined by allele profiles in the nine housekeeping genes. Occasional isolates with virulence gene alleles deviating from the majority of isolates belonging to the same ST (ST20 and ST53) were indicated by an A added to the ST designation.
