# Supplementary material for: Population Genetic Analysis of Propionibacterium acnes Identifies a Subpopulation and Epidemic Clones Associated with Acne
Source: PLoS One. 2010 Aug 19;5(8):e12277. doi: 10.1371/journal.pone.0012277 (PMC2924382; doi:10.1371/journal.pone.0012277)
Supplement: Table S3 — Phenotypic characteristics of P. acnes isolates assigned to each phylogenetic division. (0.03 MB DOC) [file pone.0012277.s006.doc]

**Table S3.** Phenotypic characteristics of *P. acnes* isolates assigned to each phylogenetic division

| Genetic division | Percentage of positive strains^1^ | | | | | | | | |
| --- | --- | --- | --- | --- | --- | --- | --- | --- | --- |
|  | N | Neura-  minidase | α-glucosidase | Indole | Hyaluro-nidase | Hemolysis | Ribose ferm. | Erythritol ferm. | Sorbitol ferm. |
| I-1a | 77 | 100 | 15^2^ | 100 | 0 | 64^3^ | 97 | 30 | 89 |
| I-1b | 13 | 50 | 0 | 100 | 0 | 100 | 100 | 85 | 38 |
| I-2 | 23 | 0 | 75 | 92 | 100 | 100 | 100 | 96 | 93 |
| II | 26 | 0 | 66 | 93 | 100 | 0 | 46 | 23 | 0 |
| III | 4 | 0 | 0 | 100 | 0 | 0 | 0 | 0 | 0 |

^1^ Percentages were calculated on the basis of tests performed on all 143 independent isolates.

^2^ Strains positive for α-glucosidase belonged to ST27.

^3^ All 11 isolates of the cluster composed of STs 1-4, in addition to 10 isolates of ST18, and 5 isolates assigned to ST5, ST14, ST17, and ST25 were hemolysis negative.
