# Supplementary material for: Population Genetic Analysis of Propionibacterium acnes Identifies a Subpopulation and Epidemic Clones Associated with Acne
Source: PLoS One. 2010 Aug 19;5(8):e12277. doi: 10.1371/journal.pone.0012277 (PMC2924382; doi:10.1371/journal.pone.0012277)
Supplement: Table S4 — P. acnes strains from public collection included in the study and their assignment to genetic divisions and sequence type (ST). (0.06 MB DOC) [file pone.0012277.s007.doc]

**Table S4.** *P. acnes* strains from public collection included in the study and their assignment to genetic divisions and sequence type (ST).

| **Strain** | **Isolated from** | **Isolation**  **place** | **Isolation**  **time** | **Genetic**  **division** | **ST** |
| --- | --- | --- | --- | --- | --- |
| NTCC 737/LGM16711 | Facial acne | London | 1920 | I-1a | 18 |
| CCUG 1794 | Facial acne | London | 1920 | I-1a | 18 |
| CCUG 6369 | Subcutaneous abscess | USA | 1952 | II | 52 |
| CCUG 6528 | Acne | Sweden | 1977 | II | 52 |
| CCUG 10171/ LMG16712 |  | UK | 1980 | I-1a | 27 |
| CCUG 27534 | Urinary tract | London | 1979 | II | 51 |
| CCUG 32901 | Blood | Sweden | 1994 | I-2 | 33 |
| CCUG 33192 | Wound | Sweden | 1994 | I-1a | 18 |
| CCUG 33206/ LMG16715 | Blood | Sweden | 1994 | II | 57 |
| CCUG 33950 | CSF | Sweden | 1995 | II | 53 |
| CCUG 33951 | Blood | Sweden | 1995 | II | 48 |
| CCUG 34938 | Blood | Sweden | 1995 | I-1a | 19 |
| CCUG 35547 | Oliocranian bursitis | Sweden | 1996 | III | 43 |
| CCUG 35749 | Hip prostesis | Norway | 1996 | III | 43 |
| CCUG 35900 | Biopsy | Sweden | 1996 | III | 43 |
| CCUG 36609 | Human pustules | Sweden | 1996 | II | 53 |
| CCUG 36661 | Blood | Sweden | 1996 | I-2 | 42 |
| CCUG 36986 | Wound re-operated hip | Sweden | 1996 | III | 44 |
| CCUG 37286 | Blood | Sweden | 1996 | II | 52 |
| CCUG 38203 | Blood | Norway | 1997 | I-1a | 18 |
| CCUG 38293 |  | Sweden | 1997 | II | 52 |
| CCUG 38453 | Femur biopsy | Sweden | 1997 | I-1a | 27 |
| CCUG 38584 |  | USA | Before 1963 | I-1a | 20 |
| CCUG 45436 | Oral cavity | Sweden | 2001 | II | 55 |
| CCUG 47251 | Hip tissue | Sweden | 2002 | I-2 | 33 |
| CCUG 48138 | Synovial fluid | Sweden | 2003 | I-2 | 36 |
| CCUG 48370 | Purulent vaginal discharge | UK | 2003 | I-1a | 3 |
| CCUG 50480 | Endocarditis | Sweden | 2004 | I-1a | 6 |
| CCUG 50655 | Mandibular gland | Sweden | 2005 | II | 46 |
| DSM 16379 | Contamination | Germany |  | I-2 | 34 |
