# Supplementary material for: Population Genetic Analysis of Propionibacterium acnes Identifies a Subpopulation and Epidemic Clones Associated with Acne
Source: PLoS One. 2010 Aug 19;5(8):e12277. doi: 10.1371/journal.pone.0012277 (PMC2924382; doi:10.1371/journal.pone.0012277)
Supplement: Table S5 — Genes identified as missing in one of the complete genomes of P. acnes strains KPA171202 and SK137 and their presence in the genomes of three additional genomes representing distinct evolutionary clusters. (0.07 MB DOC) [file pone.0012277.s008.doc]

**Table S5.** Genes identified as missing in one of the complete genomes of *P. acnes* strains KPA171202 and SK137 and their presence in the genomes of three additional genomes representing distinct evolutionary clusters.

| I-1a | | | I-2 | II | Putative function |
| --- | --- | --- | --- | --- | --- |
| SK137 | SK187 | J165 | KPA171202 | SK139 |
| 3127-3133 | - | - | - | - | ABC transporter, recombinases, N-acetylmuramoyl-l-alanine amidase, hypotheticals |
| 3169-3171 | - | - | - | - | Hypotheticals |
| 3172-3178 | - | - | - | - |  |
| 3179-3192 | - | - | - | - | CAAX amino terminal protease family protein, YcaO-like protein, SagB-type dehydrogenase domain protein, ABC transporters, hypotheticals |
| - | - | - | PPA2367 | - | Hypothetical |
| - | + | + | PPA294 | + | PTS system sugar-specific EII component |
| - | - | - | PPA295-299 | + | Putative sugar transporter, 4-hydroxythreonine-4-phosphate dehydrogenase 2, Deo family transcriptional regulator |
| - | - | - | PPA0372-382 | + | Hyaluronate lyase, glycosyl hydrolase, oxidoreductase, 3-ketoacyl-reductase |
| 3411-3412 | - | - | - | - | Kinase, pfkB family, 4-hydroxy-2-oxoglutarate aldolase |
| - | - | - | PPA2398 | - | Hypothetical |
| 3457-3459 | - | - | - | - | Hypotheticals |
| - | - | - | PPA738-739 | + | Gluconate permease, glukonokinase |
| - | + | - | *PPA846-74, PPA2354, PPA2400, PPA2355 | - | ABC transporters, conjugal transfer system, lanthionine biosynthesis, transposases |
| 3911, 3913 | - | - | - | - | Hypotheticals |
| 3966 | + | + | - | - | Acetyl-coenzyme A synthetase family protein |
| 4130 | + | + | - | + | Polyphosphate:nucleotide phosphotransferase, PPK2 family |
| 4210 | - | + | - | - | Hypothetical |
| - | - | - | PPA2384  PPA2383 | - | Hypotheticals |
| - | + | + | *PPA1278-1304 | + | Plasmid partition protein, biotin carboxylase, thioesterase, non-ribosomal peptide synthetase, surfactin synthetase, biosurfactants production protein |
| 4518-4528 | + | + | - | - | Glycosyl hydrolase, Tat (twin-arginine translocation) pathway signal sequence, ABC transporters, hypotheticals |
| 4648 | + | + | - | - | Hypothetical |
| - | - | - | *PPA1579, 1583-1613 | - | Prophage |
| 4712 | + | + | - | - | Hypothetical |
| 5001-5004 | + | + | - | + | ABC transporters, hypotheticals |
| 5128-5131 | + | + | - | + | Cupin domain protein, hypotheticals |
| 5142 | - | + | - | - | Hypothetical |
| - | - | - | *PPA2055-70 | - | β-glucanase, α-galactosidase, α-L-fucosidase precursor, sugar-binding protein, permeases, LacL family transcription regulator |
| - | - | - | *PPA2081-7 | - | ABC transporters and permeases |
| - | + | + | PPA2119 | + | Hypothetical |
