# Supplementary material for: Population Genetic Analysis of Propionibacterium acnes Identifies a Subpopulation and Epidemic Clones Associated with Acne
Source: PLoS One. 2010 Aug 19;5(8):e12277. doi: 10.1371/journal.pone.0012277 (PMC2924382; doi:10.1371/journal.pone.0012277)
Supplement: Table S6 — Primers used for amplification and sequencing of P. acnes gene loci. (0.04 MB DOC) [file pone.0012277.s009.doc]

**Table S6.** Primers used for amplification and sequencing of *P. acnes* gene loci.

| Locus | Primer sequences | Coordinates in gene | Size (bp) of trimmed  fragment | Reference |
| --- | --- | --- | --- | --- |
| *camp5* | F-5´CCACGCCATGAGCTAAGGACAG 3´  R-5´TGAACTAGACCGCGGCAAACATT 3´ | 1- 834 | 834 | Valanne *et a*l., 2005 |
| *cel* | F-5’ GCC GAC GTT TTC TAC AGT GAG C 3’  R-5’ GGC GGT GAG GGT CCA TTC A 3’ | 280 - 642 | 363 | www.mlst.net |
| *coa* | F- 5’ GCG GGA ATC GAG GGT GCT A 3’  R-5’ AGG GCC GCC GCT AGA TAA GTA 3’ | 226 - 753 | 528 | www.mlst.net |
| *fba* | F-5’ AGG ACC CGC TAT TTC AAC TCT CA 3’  R-5’ ACG CGG GTC GTA CAT CTT CTT 3’ | 379 - 891 | 513 | www.mlst.net |
| *gms* | F-5’ CCG CCT CAC CGT CCA GCA 3’  R-5’ CAC ATC GAG AAC CGC ATC ACTC 3’ | 493 - 966 | 474/462 * | www.mlst.net |
| *lac* | F-5’ GCC GCA GCC TTG GGA CTC T 3’  R-5’ GAA ATG CTG TCG CCC CGT G 3’ | 109 - 528 | 420 | www.mlst.net |
| *oxc* | F-5’ GTG CTG CCG GAA AAG TCG 3’  R-5’ CAC CGG CGT CAG GAT TGT 3’ | 72 - 444 | 372 | www.mlst.net |
| *pak* | F-5’ CGACGC CTC CAA TAA CTT CC 3’  R-5’ GTC GGC CTC CTC AGC ATC 3’ | 286 - 678 | 393 | www.mlst.net |
| *recA* | F-5´AGCTCGGTGGGGTTCTCTCATC 3´  R-5´GCTTCCTCATACCACTGGTCATC 3´ | 115 - 921 | 807 | McDowell *et al.*, 2005 |
| *tly* | 5´CAGGACGTGATGGCAATGCGA 3´  5´TCGTTCACAAGACCACAGTAGC 3´ | 1 - 777 | 777 | McDowell *et al.*, 2005 |
| *zno* | 5’ CGC CGG CAT CAC CAC CTA TT 3’  5’ TCT CAC ATC GCC CGC AAC C 3’ | 520 - 936 | 417 | www.mlst.net |

* 12 bp in-frame deletion in CCUG35749, CCUG35900 and CCUG36986
